# Hyaluronic acid-solid lipid nano transporter serum preparation for enhancing topical tretinoin delivery: skin safety study and visual assessment of skin

**DOI:** 10.3389/fphar.2024.1401594

**Published:** 2024-09-26

**Authors:** Yasir Mehmood, Hira Shahid, Syeda Momena Rizvi, Usama Jamshaid, Numera Arshad, Mohammad Nur-e-Alam, Muhammad Delwar Hussain, Mohsin Kazi

**Affiliations:** ^1^ Riphah Institute of Pharmaceutical Sciences (RIPS), Riphah International University Faisalabad, Faisalabad, Pakistan; ^2^ Department of Pharmacology, Faculty of Pharmaceutical Sciences, Government College University Faisalabad, Faisalabad, Pakistan; ^3^ Department of Biochemistry, University of Agriculture, Faisalabad, Punjab, Pakistan; ^4^ Faculty of Pharmaceutical Sciences, University of central Punjab, Lahore, Pakistan; ^5^ Department of Pharmacy, COMSATS University Islamabad, (Lahore Campus), Islamabad, Pakistan; ^6^ Department of Pharmacognosy, College of Pharmacy, King Saud University, Riyadh, Saudi Arabia; ^7^ Department of Pharmaceutical Sciences, School of Pharmacy, University of Maryland Eastern Shore, Princess Anne, MD, United States; ^8^ Department of Pharmaceutics, College of Pharmacy, King Saud University, Riyadh, Saudi Arabia

**Keywords:** hyaluronic acid, filler, topical, solvent, diffusion, tretinoin

## Abstract

The use of nanosized particles is becoming more popular for the topical treatment of skin conditions. In this research work, we created and investigated the effects of solid lipid nanoparticles (SLN) containing hyaluronic acid and tretinoin. Solvent emulsification diffusion method was used to prepare the SLN formulation and characterized for their physicochemical properties. Fourier transform infrared spectroscopy was used to confirm that hyaluronic acid and tretinoin were incorporated in the SLN. Furthermore, X-ray diffractogram, thermal analysis including DSC and TGA and *in vitro* dissolution, permeation tests were also performed along with microbial assessment. Clinical studies in human were performed to evaluate the effect of the SLN on skin wrinkles. The SLN was 750 ± 31.29 nm in size, with a zeta potential of 13.07 ± 0.75 mV and a narrow polydispersity index of 0.24 ± 0.12. The entrapment efficiency of tretinoin was found to be 90.07 ± 4.79%. Clinical studies in healthy human volunteers demonstrated that 90% of the tested individuals had improved skin conditions (reduction in wrinkles), by at least one grade after 4 weeks of treatment. Regarding the development on SLN, it was found that the internal phase concentration did not considerably affect the physicochemical and microbiological properties. Therefore, Hyaluronic acid has potential for the development of SLN applicable to cosmetic formulations, especially for skin. These findings show that the developed SLN have potential for use as cosmetics in the future.

## Introduction

Tretinoin (TR), also known as all-trans retinoic acid, is a vitamin A metabolite used topically to treat a variety of skin conditions, including acne and wrinkles or skin aging (Zasada and Budzisz, 2019; Szymański et al., 2020; Riahi et al., 2016). However, a number of drawbacks, including skin irritability and high chemical instability, restrict its application (Raminelli et al., 2018; Keeling et al., 2008). Tretinoin, chemically named (2E,4E,6E,8E)-3,7-dimethyl-9-(2,6,6-trimethylcyclohex-1-enyl)nona2,4,6,8-tetraenoic acid, is a dermatological agent. It is found as a yellow or light orange crystalline powder that is only slightly soluble in alcohol and nearly insoluble in water. In particular, it is sensitive to heat, light, and oxygen when in solution. It decomposes and melts at roughly 182℃. The chemical formula of tretinoin is C_20_H_28_0_2_, and its molecular mass is 300.44 g mol^−1^.

Topical therapy of skin conditions seems preferable due to the lower likelihood of systemic side effects (Schäcke et al., 2009; Schäfer-Korting et al., 2007). However, the stratum corneum prevents xenobiotics from penetrating living skin, and only a small percentage of the applied medication can be absorbed. Dermal penetration can be increased by using particle carrier systems (Prow et al., 2011). Skin lipids play a significant role in the penetration barrier and lipid carriers that could facilitate lipid exchange with the skin’s surface (Zhai and Zhai, 2014). Lipid microspheres, solid lipid nanoparticles (SLN), nanostructured lipid carriers (NLCs), liposomes, microemulsions and hexagonal phase nanodispersions are some examples of carrier systems for skin surfaces (Müller et al., 2002; Montenegro et al., 2016). For topical SLN, all excipients used in current topical cosmetic and dermal pharmaceutical products can be used (Müller et al., 2002). There are several cosmetic product are now marketed with nano technology (Jose and Netto, 2019).

In fact, the type of lipid particle and the way that drugs interact with the lipid matrix were found to be important. Drug skin penetration can be improved by SLN. SLN can also cause epidermal targeting, particularly when the drug is at the particle surface. In this study we have used TR for wrinkles treatment along with Hyaluronic acid (HA). HA is a naturally occurring polymer composed of unbranched repeating units of glucuronic acid and N-acetyl glucosamine linked by 1–3 and 1–4 glycosidic bonds (Kong et al., 2011). HA is a biopolymer that has found extensive usage in the pharmaceutical industry due to its array of intriguing characteristics, including bioadhesion (ability to stick to negatively charged surfaces such as skin and mucosa). It is a part of the extracellular matrix and is also found in connective, epithelial, and brain tissues. Hyaluronic acid serves as a skin conditioner and/or a viscosity modifier in cosmetic compositions. HA is mostly found in anti-aging cosmetics (Juncan et al., 2021). HA is still a relatively new chemical compound of treating wrinkles and lines on the face, and it has already made significant strides in the field of cosmetic surgery. Hyaluronic acid fillers have been given the go-ahead by the Food and Drug Administration, as well as their current use in facial cosmetics (Monheit and Coleman, 2006). Under physiological circumstances, HA may also work in the skin as an antioxidant and free radical scavenger (Pavicic et al., 2011). To enhance contact and internalization in ocular cells, solid lipid nanoparticles have been combined with HA. The bioadhesive ability of HA can improve drug retention at the application site.

In this project, transdermal administration of SLN with HA and TR (H-T-SLN) was studied for biological applications to treat wrinkles. Due to the synergistic effects of the following elements, HA may be the essential component that helps Nano constructs penetrate the skin more effectively. The hygroscopic HA molecules moisten skin tissues and enable conjugated cargoes to go through the skin transdermally. In this research, we used HA as SLN vehicle and it is most promising material for skin therapeutic purposes.

We aimed to prepare HA and tretinoin-associated SLN. The nanoparticle size, zeta potential, polydispersity index (PDI), association efficacy, loading efficacy, structural confirmation, shape and safety are among its many physicochemical properties. A range of biophysical approaches can be used to objectively and noninvasively measure the ageing of the skin in response to topical treatments. Moreover, we prepared serum by using loaded SLN for customer use and examined human skin by using a camera Visioscan^®^ camera. In the current investigation, a 4-week phase II single controlled group, before-after clinical study was developed and investigated to determine the anti-wrinkle impact of a topical H-T-SLN serum comprising HA and tretinoin on a variety of skin aging endpoints.

## Materials and methods

### Chemicals

Saffron Pharmaceuticals gratefully offered tretinoin (Faisalabad, Pakistan). Dialysis membranes with a molecular weight cut-off (MWCO) of 8,000 Da were purchased from Spectrum Medical Industries (Houston, Texas, United States). Dae-Jung (City, Korea) chemicals were used to obtain ethanol, glycerin, propylene glycol, polylobate 80, cetostearyl alcohol and hyaluronic acid. We purchased hyaluronic acid from Merck (CAS No.: 9012-76-4). 2-Phenylethanol (Epikuron) was purchased from Lucas Meyer (Hamburg, Germany). Double-distilled water produced at a nearby plant was utilized throughout the trials and conductivity of water was (2.1 *μs/cm* ^2^). All of the other chemicals and solvents were analytical grade, and we used them exactly as they were provided.

### Preparation of SLN

**FIGURE 1:**
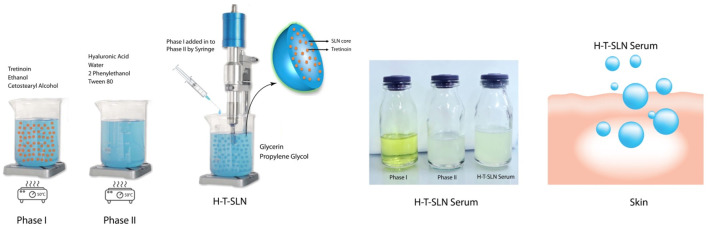
*Preparation method of* H-T-SLN serum is mentioned Phase I (oil phase) and Phase II (water phase).

The solvent emulsification diffusion method was used to create SLN. Emulsifiers (cetostearyl alcohol) were dispersed in 10 mL distilled water with magnetic stirring at 50 ℃ . After 15 min, ethanol was added dropwise to this dispersion (10 g). Ten milligrams of tretinoin was mixed by continuous stirring. This is known as Phase I (oil phase). Phase II (water phase) was prepared by adding hyaluronic acid in water (15 mL). After 30 min of continuous stirring, Tween 80 and 2-phenylethanol (fragrance agent) were added in this water phase. After this, phase I (oil phase) was added into phase II (water phase) by using a 24 gauge needle syringe and probe sonicator (JY92-2D; Xinzhi Equ. Inst., China) (300 W). The probe was sonicated for 20 min at 40% amplitude and then homogenized for 5 min at 12,000 rpm. Hyaluronic acid tretinoin SLN (H-T-SLN) were filtered through a 0.45 *μ*m membrane to eliminate impurity materials (such as metal) introduced during ultrasonication, and then 10.86% glycerin and 5.43% propylene glycol were added for the formation of serum. This project was completed in a saffron pharmaceuticals industry, and saffron-developed plant capabilities allow us to support large-scale manufacturing. During lab-scale preparation, we also determined critical quality attributes and critical process parameters, which were monitored at the scale-up. Optimized formulation mentioned in Table 1 with percentage of ingredients. Optimised process for preparing mentioned in Figure 1.

**TABLE 1:** Optimized formulation of the product.

Sr. #	Material name	Quantity (g) of material	Percentage (%) of material
1	Hyaluronic acid	0.5	1.1
2	Tretinoin	0.01	0.02
3	Cetostearyl alcohol	0.5	1.1
4	Ethanol	10	21.73
5	2-Phenylethanol	2.5	5.43
6	Water	25	54.34
7	Propylene glycol	2.5	5.43
8	Glycerin	5	10.86
Total		46.01	100

2-Phenyethanol.

### Determination of entrapment efficiency

Hyaluronic acid tretinoin SLN (H-T-SLN) serum precipitation was performed by centrifuging 1 mL of loaded tretinoin nanodispersion in dichloromethane at 2,000 rpm for 15 min. The supernatant was discarded, and 500 *μL* dichloromethane was used to dissolve the silt containing tretinoin. Carry out the procedure in subdued light. The material was centrifuged for 15 min (30℃), and dichloromethane was added to a volume of 50 mL. Again, 3 mL of solution was diluted to 100 mL with a solution (prepared by diluting 5 mL of 0.1 M hydrochloric acid to 250 mL with 96% ethanol) and filtered with 0.2 *μm* Sartorius. The absorbance of this solution was measured at a maximum of 295 nm using ethanolic hydrochloric acid solution as a blank. The content of tretinoin was calculated by taking 1,350 as the value of a (1% 1 cm) (Quartz cells) at 295 nm. The acquired absorbance was compared to the standard, and a triplicate calculation of the drug loading efficiency was made. Equation 1 was used to determination of entrapment efficiency of particles (Karami and Abdouss, 2024; Yang et al., 2024).

$$\text{Entrapment }\text{efficiency }\left(\%\right)=\frac{Drug\;added-Free\;drug}{Drug\;added}\;X\;100$$ (1)

### Particle size, polydispersity index (PDI) and zeta potentials

Photon correlation spectroscopy (PCS) (Nano ZS Zetasizer, Malvern Instruments Corp, United Kingdom) was used to measure the mean diameter (Z-average diameter), polydispersity and size distribution at 25°C in polystyrene cuvettes with a path length of 10 mm. Using a Nano ZS Zetasizer and laser Doppler anemometry, the zeta potential was calculated. Deionized water from a Milli-Q system was used for the measurements, which were carried out in capillary cells with 10 mm path lengths. A triple of each measurement was made (Mehmood et al., 2020).

### FTIR spectroscopy

*A* Thermo Scientific Nicolet iN5 FTIR United States with diamond ATR was used in the current experiment. A small sample was added to the apparatus with the use of a spatula, and the fixing clamp was tighter. In the 800–4,000 cm^−1^ region, this method was employed to capture the spectra of tretinoin, hyaluronic acid and hyaluronic acid tretinoin SLN (H-T-SLN) (Ruther, 2022).

### Thermal analysis DSC and TGA study

Calorimetry experiments were carried out using DSC and TGA equipment (Mettle Toledo DSC823, Switzerland). Tretinoin, Hyaluronic acid and Hyaluronic acid Tretinoin SLN (H-T-SLN) were all measured, with an empty aluminum pan serving as the standard. In 40 μL aluminum pans, 1–2 mg of sample was precisely weighed, and the DSC/TGA spectrum was captured while the temperature was heated at a rate of 5 K/min from 25 to 300℃. Each experiment was carried out three times (Nasrollahi et al., 2013).

### XRPD study

*An* X-ray powder diffraction analyser manufactured by DADVANCE, Bruker, Germany was used to examine Tretinoin, Hyaluronic acid and Hyaluronic acid Tretinoin SLN (H-T-SLN). The instrument was run at 30 kV and 15 mA with Cu Kα radiation (1.542 Å) in the range of 5 to 40 2θ. The information was then computed as peak height (intensity) versus 2θ (Mehmood et al., 2019).

### Morphology study

An examination of morphology was performed using a scanning electron microscope (JEOL Ltd., Tokyo, Japan). We used gold-coated dual-sided adhesive tape with dry samples, Tretinoin in crystal form and Hyaluronic acid Tretinoin SLN (H-T-SLN) were analysed (magnification: 30,000×; accelerating voltage: 20.0 kV). Analysis was performed at 25°C ± 2°C (Smirnova et al., 2023).

### *In vitro* release study

Utilizing the dialysis membrane method, *in vitro* research on tretinoin, hyaluronic acid and hyaluronic acid tretinoin SLN (H-T-SLN) was conducted. The SLN equivalent of 1 mg was diluted in 5 mL of distilled water and placed inside a dialysis bag (Spectrum Medical Industries, Houston, Texas, United States). A closed pouch was created by sealing the bag. The dialysis bag was then suspended separately in 500 mL of medium that was kept at 37°C with constant stirring at 50 rpm and contained phosphate buffer with a pH of 7.4 and 0.1 M HCl with a pH of 1.2 at different time points. The 2.0 mL samples were taken out and refilled at the same volume from new media under the same conditions at predetermined intervals up to 60 min. The resultant filtrate was collected through a 0.45 m pore size filter and used for UV analysis after being diluted (Zhang et al., 2010). The percentage release was calculated by following Equation 2 (Barkat et al., 2018; Khalid et al., 2018; Mehmood et al., 2023a):

$$Drug\;release\;percentage=\frac{sample\;absorbance}{standard\;absorbance}\times 100$$ (2)

### Hemolytic investigations

Hyaluronic acid Tretinoin SLN (H-T-SLN) was mixed with rat blood to determine the hemolytic investigation. Permission was obtained from the ethical review board of Rashid Latif College of Pharmacy (RLCP) Lahore, Pakistan IRB No (RLCP/EP/28/2022). A tube containing ethylene diamine tetraacetic acid was used to collect blood for the hemolytic test, and after 5 min of centrifugation at 1,500 rpm, the supernatant was removed, while the precipitate was washed three times with phosphate-buffered saline (PBS). Then, 200 *μL* of washed blood was mixed with 3.8 mL of phosphate-buffered saline and vortexed for a while. The mixture was centrifuged at 1,600 rpm for 5 min after the samples were kept at 37°C for 2 h. Measure the absorbance of the supernatant at 541 nm. In this experiment, phosphate-buffered saline was chosen as the negative control, and Triton-X was chosen as the positive control. The malformation of blood cells was observed through microscopy (not shown in article), and % hemolysis was determined by using Equation 3 (Mehmood et al., 2023b; Mehmood et al., 2023c).

% Hemolysis=ABS−ABS0/ABS100−ABS0 ∗ 100% (3)

### *In vitro* diffusion studies

For the diffusion study of Hyaluronic acid Tretinoin SLN (H-T-SLN), Franz Diffusion Cell (local made by IPS Analytical system, Lahore, Pakistan) was used. A silicon membrane (Silatos™ 0.13 mm silicone, Teleflex Medical, Westmeath, Ireland) was used in this experiment. The penetration of the H-T-SLN was evaluated using a vertical Franz diffusion cell with a donor surface area of 1.2 cm^2^ and a receptor volume of 5.2 mL with a silicon membrane. A semisynthetic Silatos™ 0.13 mm silicone membrane was adjusted within the donor and receptor chambers of the Franz diffusion cell. In a 1.0 mL volume, the H-T-SLN sample was added. The temperature of the receptor chamber was maintained at 37°C while phosphate buffer (pH 7.4) was circulated. The membrane and receptor phase were in contact for 1 h at a temperature of 37 0.5°C before the experiment. Samples (0.5 mL) were taken every time for 120 min (5, 20, 40, 60, 80, 100 and 120 min) during the receptor phase, and 50 mL of dichloromethane was added to the samples at predefined intervals. Once more, 3 mL of solution was diluted in 100 mL of solution (5 mL of 0.1 M hydrochloric acid in 250 mL of 96% ethanol). Using ethanolic hydrochloric acid solution as a blank, the absorbance of this solution was measured at 295 nm. The amount of tretinoin was determined by using the value of a (1% 1 cm) 1,350 at 295 nm (Mehmood et al., 2019; Thakur et al., 2020; Abdul et al., 2020; Jacob et al., 2020).

### Skin safety evaluation

According to a prior report, a skin safety evaluation was conducted (Im et al., 2020). In summary, skin safety testing involved determining skin irritation through a medical exam and interview after 1, 2, and 4 weeks of product use (Im et al., 2020).

### Physical stability and microbial assessment

The prepared Hyaluronic acid Tretinoin SLN (H-T-SLN) was kept in accelerated stability studies (40°C ± 2°C/75% ± 5% RH) for 6 months (Table 2). A physicochemical analysis including description, odor, pH, particle size, PDI, viscosity and microbial assessments was performed in the initial, 3rd and 6th months.

**TABLE 2:** Stability results until 6 months with 40 ℃

Test	Results			Limits
	Initial	3rd Month	6th Month	
Order	Complies	Complies	Complies	Characteristic order
Description	Complies	Complies	Complies	Clear Yellowish
Particle size	**750 ± 31.29**	Nil	**710 ± 41.12**	NA
pH	**6.5**	**6.3**	**6.5**	6–6.5
PDI	**0.24 ± 0.12**		**0.27 ± 0.22**	NA
Viscosity	**80cpi**	**83cpi**	**79cpi**	75–85cpi
Total Aerobic Microbial Count (TAMC)	<10 cfu/mL	<10 cfu/mL	<10 cfu/mL	200 cfu/mL
Total Combined Yeasts & Molds Count (TYMC)	<10 cfu/mL	<10 cfu/mL	<10 cfu/mL	20 cfu/mL
Tests for Absence of specified Microorganisms				
*Staphylococcus aureus*	Absent/mL	Absent/mL	Absent/mL	Should be absent/mL
*Pseudomonas aeruginosa*	Absent/mL	Absent/mL	Absent/mL	Should be absent/mL

^a^ Nil means no limits were determined after 3^rd^ month.

### Study design

Hyaluronic acid tretinoin SLN (H-T-SLN) serum antiaging efficacy on face skin was examined in a 4-week, single-group clinical experiment (Female and Male) (13) Between September 2022 and October 2022, the research was conducted at the RLCP. The Rashid Latif College of Pharmacy and Medical College’s institutional review board authorized the protocol in accordance with the rules established by the faculty of pharmacy at the college. Approval: IRB No (RLCP/EP/30/2022). Additionally, the study was conducted in compliance with the ethical standards of the Declaration of Helsinki and Good Clinical Practice (GCP). Before collecting their signed informed consent, all volunteers were taken of the study’s approach. The participants were told to apply one fingertip unit of the H-T-SLN Serum on their face every day for 4 weeks. Consent was taken from all participants and informed them about all experiments.

### Inclusion and exclusion criteria

The following requirements were established as inclusion standards:

1. Good physical and health condition.
2. Age 40–60
3. Volunteer (Female and Male) who completed the written agreement form used anti-wrinkle H-T-SLN serum once daily for 4 weeks and underwent regular follow-up.

The prerequisites for exclusion were as follows:

1. Using injectable fillers, anti-aging therapies, or systemic or topically applied wrinkle creams within the last 12 months.
2. Previous 3-month history of isotretinoin use.
3. Over the past 6 months, there have been interventions such as laser therapy or comprehensive chemical peeling.
4. The existence of severe or cystic acne on the face.
5. Hypersensitivity issues.
6. Women who are expecting or nursing.

### Visual evaluation of wrinkles in the skin

The procedure for evaluating skin wrinkles visually was carried out as previously mentioned (Im et al., 2018). In brief, the evaluation of winkles around the eyes was performed under specific lighting conditions. The evaluation was conducted by two evaluators using specified standards. Wrinkles were analysed and recorded at the end of the study. The mean evaluations for each assessor were generated and statistically examined (Table 3). During all procedures and subsequent visits, all adverse events were either reported by the subjects or observed by the researchers and noted.

**TABLE 3:** Visual evaluation of wrinkles in the skin.

Description	Grade	Subjects before (0 week)	Subject after 4 weeks
There are no skin wrinkles	1	0	0
Thin wrinkles on the skin visible	2	7	4
There are many thin wrinkles on the skin	3	2	5
Deep wrinkles on the skin are visible	4	1	1

### Evaluation of skin wrinkle parameters using Visioscan^®^ camera images

The Visioscan^®^ camera is the ideal instrument for accurately assessing deeper and larger macro wrinkles, such as crow’s feet. The measurement was performed using an oblique light source and a skin facsimile. The length, depth, and area of the wrinkles are measured in millimetres using a high-resolution camera that is positioned vertically next to the replica to transform them into digital form. Using a camera and software, the surface evaluation of living skin was assessed. Four clinical characteristics were calculated (Table 4) to provide a quantitative and qualitative description of the skin surface as an index: skin smoothness (Se), skin roughness (Se), scaliness (Se), and wrinkles (Se).

**TABLE 4:** Volunteer skin smoothness (Se), skin roughness (Se), scaliness (Se) and wrinkles (Se).

Volunteer 0 days and 4 weeks		Roughness	Scaliness	Smoothness	Wrinkles
1	Before	1.23	2.54	334.3	45.65
	After	1.12	2.74	382.4	39.89
2	Before	1.27	2.51	324.3	55.25
	After	1.19	2.54	392.4	49.89
3	Before	1.33	2.44	328.3	41.35
	After	1.21	2.84	382.4	37.29
4	Before	1.33	2.44	314.3	43.25
	After	1.10	2.90	361.4	37.19
5	Before	1.43	2.54	368.3	45.25
	After	1.42	2.88	392.4	43.99
6	Before	1.42	2.94	398.3	41.05
	After	1.32	2.92	392.4	39.99
7	Before	1.91	2.85	351.9	55.69
	After	1.90	2.94	372.3	49.19
8	Before	1.33	2.74	344.3	55.15
	After	1.32	2.82	392.4	49.29
9	Before	1.53	2.74	304.3	45.75
	After	1.42	2.94	362.4	42.80
10	Before	1.53	2.84	333.3	66.65
	After	1.50	2.94	382.4	61.12

### Analytical statistics

One-way ANOVA and Tukey’s test were used in the statistical analysis, which was performed using the GraphPad Prism v.5 program. The mean and standard deviation were used to depict the data (SD). Statistical significance was defined as a *p*-value of 0.05.

## Results and discussion

### Fabrication of SLN by the solvent emulsification diffusion method

**FIGURE 2:**
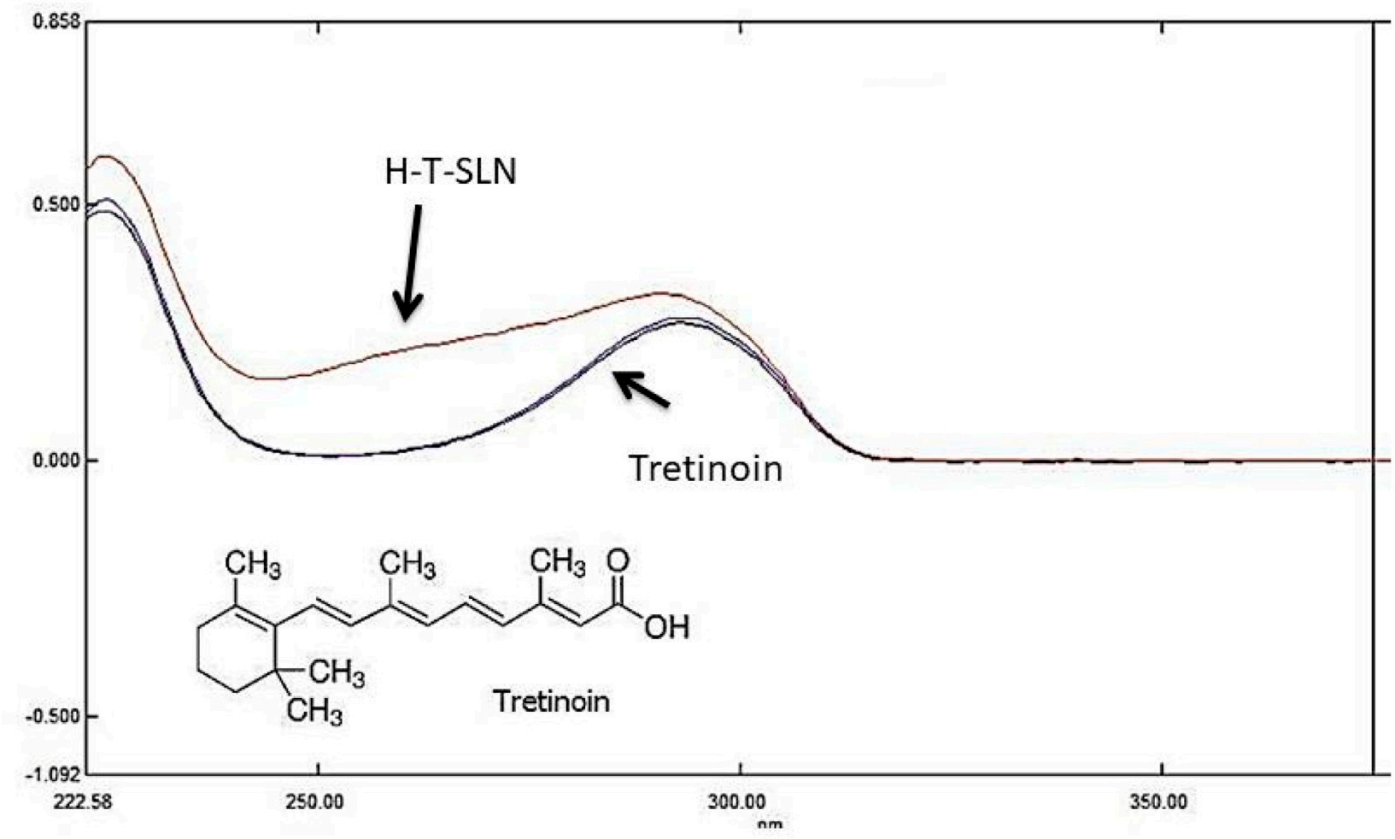
Maximum absorbance of tretinoin in UV absorbance (standard and samples (H-T-SLN) were compared at 295 nm).

Due to its relatively high cutaneous tolerability compared to other partially miscible solvents, ethanol was chosen for the current experiment as a miscible solvent for the synthesis of SLN and we have use the method as mentioned by Trotta et al. (2003). Additionally, at a temperature of 50°C, ethanol has a significant solubilizing capacity for tretinoin and cetostearyl alcohol. In this study, hyaluronic acid polymer was also used, and adding it to the SLN synthesis process made the particles more polydisperse (with a greater hydrodynamic radius). Hyaluronic acid has great potential for skin treatments. Hyaluronic acid helps skin stretch and reduces skin wrinkles and lines. The entrapment efficiency of SLN was 90.07 ± 4.79%. %. The strong affinity of the lipophilic medication for the lipidic substance, as stated by Souto et al. (2004), is likely the cause of this outcome. The production yields that were attained were typically high, ranging from 75%−80%. After the preparation of hyaluronic acid tretinoin, SLN (H-T-SLN) was converted into serum by using glycerin. Figure 2 shows the spectra of tretinoin.

### Particle size, polydispersity index (PDI) and zeta potentials

**FIGURE 3:**
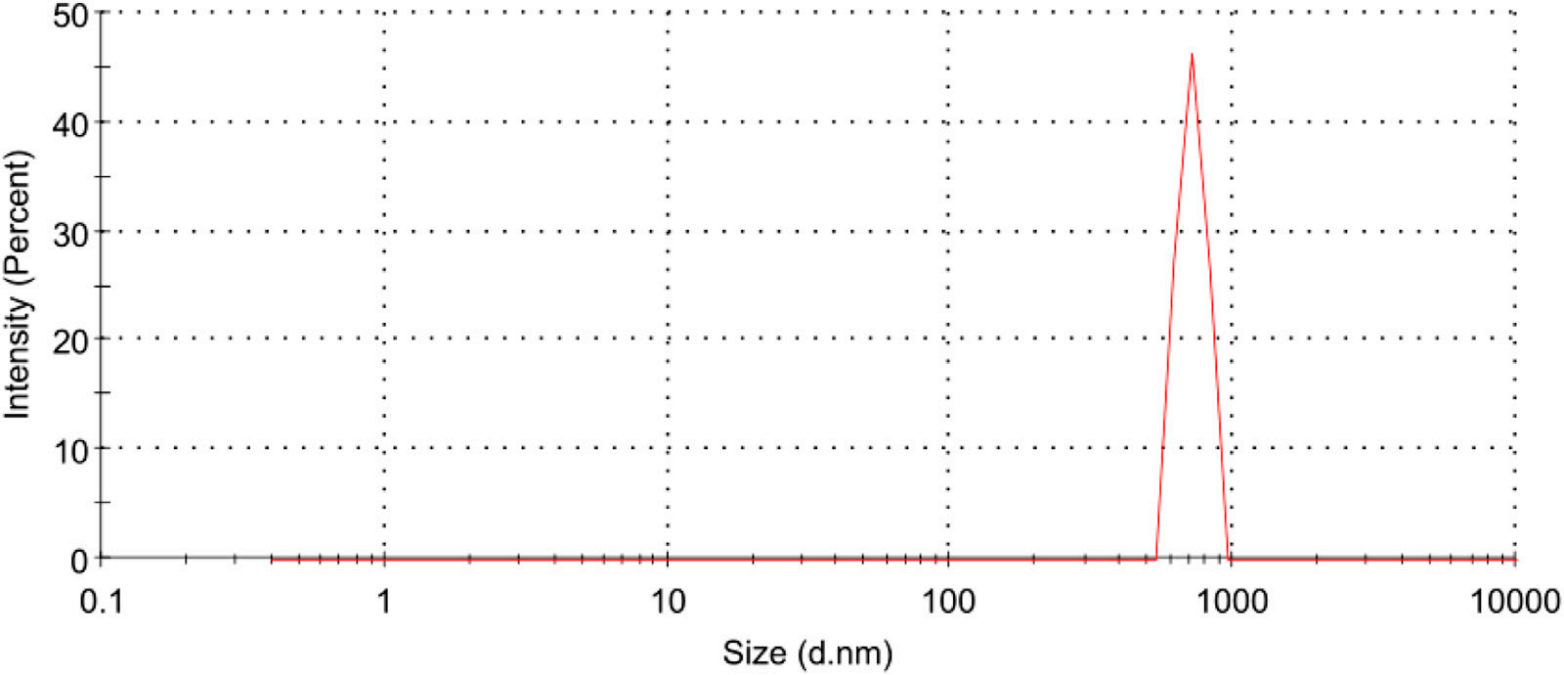
Particle size distribution measurements of (H-T-SLN) using a Malvern Instruments Zetasizer Nano ZS. Deionized water from a Milli-Q system was used for the measurements.

Hyaluronic acid tretinoin SLN (H-T-SLN), size distribution and charge were determined by using DLS. Deionized water from a Milli-Q system was used for the measurements (1 mL/10 mL). Figure 3 depicts a size distribution diagram of hyaluronic acid tretinoin SLN (H-T-SLN). The hyaluronic acid tretinoin SLN (H-T-SLN) has a hydrodynamic diameter of 750 ± 23.89** **nm and a PDI of 0.276 ± 0.03 , according to the DLS data. At a temperature of 25 ± 0.5°C, the zeta potential of the hyaluronic acid tretinoin SLN (H-T-SLN) was measured using a Malvern Zetasizer ZS200. For each sample, at least three measurements were taken. Hyaluronic acid tretinoin SLN (H-T-SLN) zeta potentials were typically approximately −16 mV, which is enough to completely stabilize the system. Same PDI was observed in the SLN formulation of Schubert and Müller-Goymann (2005).

### FT-IR analysis

FTIR spectroscopy was used in the region of 600–4,000 cm^−1^. The synthesized hyaluronic acid tretinoin SLN (H-T-SLN) was examined for the identification and characterization of functional groups present. O-H stretching vibrations are shown by the peak at 3,438 cm^−1^, C = O stretching is represented by the band at 2,901.10 cm^−1^, and the trans vinyl (CH = CH) groups of the tretinoin molecule are shown by the peak at 960 cm^−1^ (Phadtare et al., 2016).

Hyaluronic acid revealed several distinctive bands. Strong absorption bands were observed at 3,435.24 cm^−1^, which correspond to the stretching vibrations of OH. The bands at 1,632.53 cm^−1^, 1,553.51 cm^−1^ and 1,320.33 cm^−1^ can be attributed to amide, while the absorption bands at 1,155.32 cm^−1^, 1,064.12 cm^1^, 1040.67 cm^−1^ and 944.61 cm^−1^ are characteristic of carbohydrates.

**FIGURE 4:**
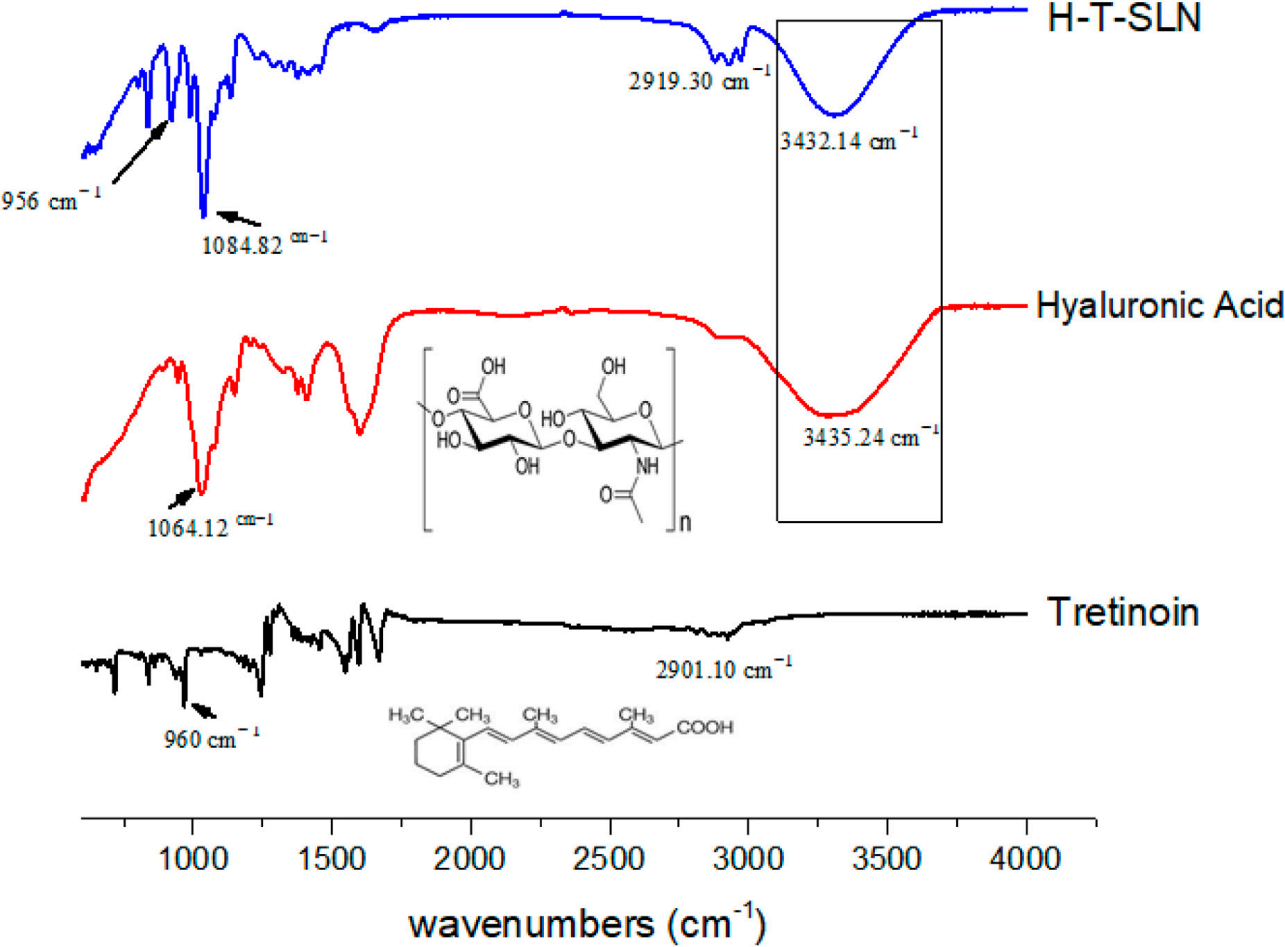
FTIR analysis of tretinoin, hyaluronic and hyaluronic acid tretinoin SLN (H-T-SLN).

For hyaluronic acid tretinoin SLN (H-T-SLN), distinctive peaks are shown in Figure 4, in which 2,919 cm^−1^ (C = O stretching of tretinoin group) was noticed, which was slightly changed from 2,901.10. The peak intensity of O-H also changed slightly, going from 3,435.24 to 3,432.14 cm^−1^. Other peaks were also found after slight shifts of 1,064.12 cm^−1^ to 1,084.82 and 960 cm^−1^ to 956 cm^−1^, which indicate that the carbohydrate group from hyaluronic acid and vinyl group from tretinoin, were present under the same conditions. It can be concluded that there was minimal interaction between tretinoin and hyaluronic acid polymer, as evidenced by the change in peak intensity, which was a sign of chemical and physical changes occurring during the SLN formation process. This interaction resulted in a high entrapment efficiency of tretinoin into the polymer.

### Thermal analysis

**FIGURE 5:**
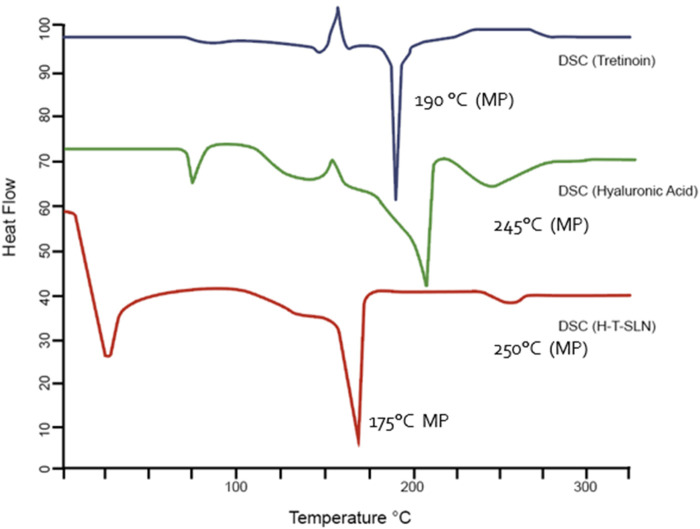
Thermal analysis (DSC) of pure tretinoin, hyaluronic acid polymer and (H-T-SLN).

Thermal analysis (Q-2000, TA, United States) was used to evaluate tretinoin, hyaluronic acid and hyaluronic acid tretinoin SLN (H-T-SLN). The temperature range was 0°C–300°C adjusted at a heating rate of 20°C/min. Figure 5 shows the DSC thermograms of tretinoin, hyaluronic acid and hyaluronic acid tretinoin SLN (H-T-SLN). The melting point of tretinoin, 194°C, was shown by a significant rise in the crystallinity of the compound. Additionally, an exothermic peak is observed at approximately 150°C, indicating an exothermic reaction caused by crystallization. Hyaluronic acid also showed an endothermic peak at approximately 55℃, an exothermic peak at approximately 150 ℃ and a final exothermic peak at 210℃ and 245°C which is melting point of Hyaluronic acid. A totally amorphous version of the polymer known as hyaluronic acid exists at a glass transition temperature (Tg) of approximately 210℃. Hyaluronic acid tretinoin SLN (H-T-SLN) displayed a broad endothermic peak at 25.4°C in contrast to tretinoin, which was due to dehydration and elimination of volatile components. A second endothermic peak was observed at 155℃, which was due to tretinoin amorphous dispersion in the lipid matrix, and this indication showed that tretinoin crystals convert into an amorphous phase that becomes more soluble.

**FIGURE 6:**
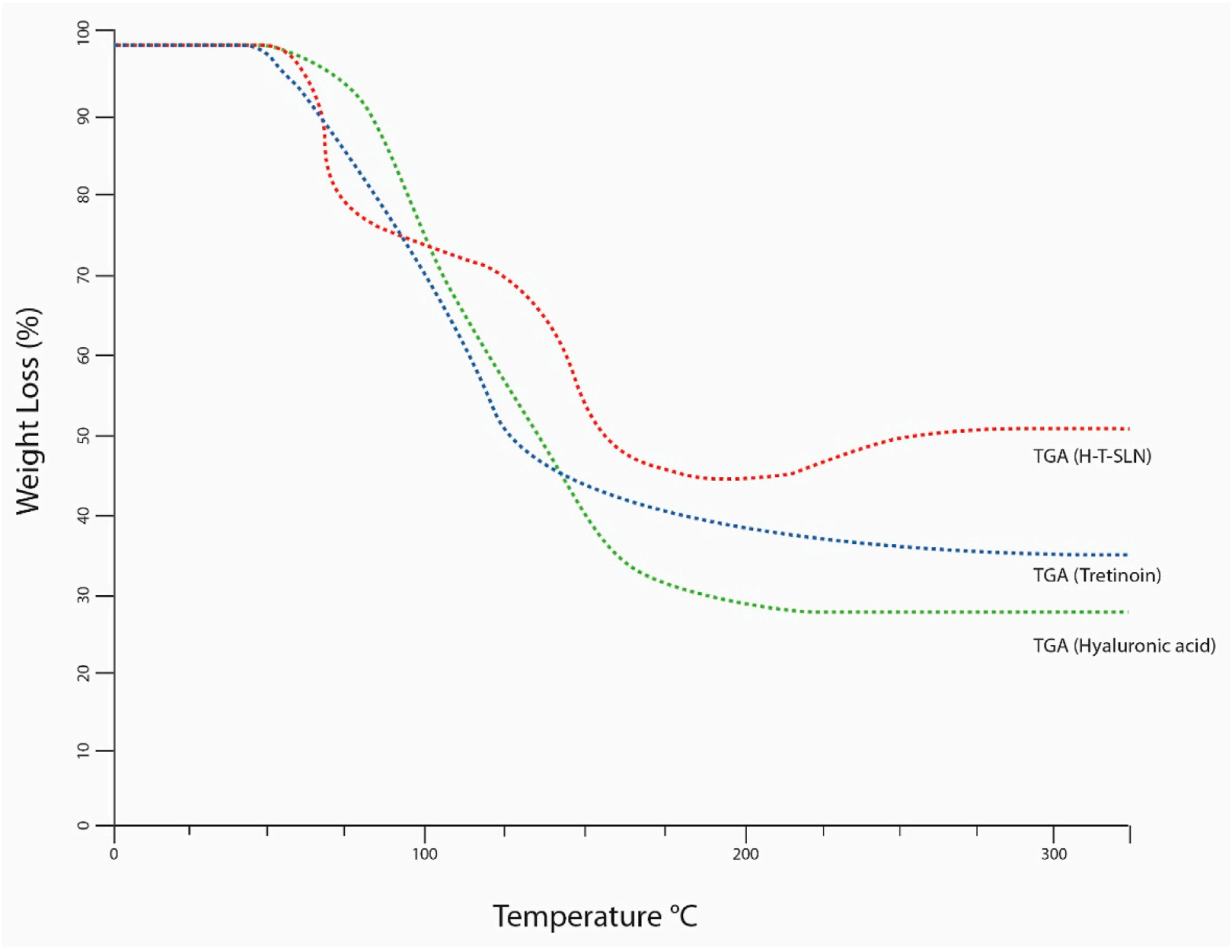
Thermal analysis (TGA) of pure tretinoin, hyaluronic acid polymer and (H-T-SLN).

The same tool and process were used to calculate weight loss. The weight loss of tretinoin began between 50°C and 120℃, and a total weight loss of 60% was observed in TGA (Figure 6). At a glass transition temperature (Tg) of approximately 175°C, the polymer known as hyaluronic acid exists in a completely amorphous state. At 175°C and 250°C, for hyaluronic acid, TGA also showed a weight loss of 70%. The weight loss of hyaluronic acid tretinoin SLN (H-T-SLN) was significantly changed due to drug and polymer interactions within lipid molecules. TGA showed a total weight loss of 40%, which started from 75℃ to 178℃.

### Morphology analysis

**FIGURE 7:**
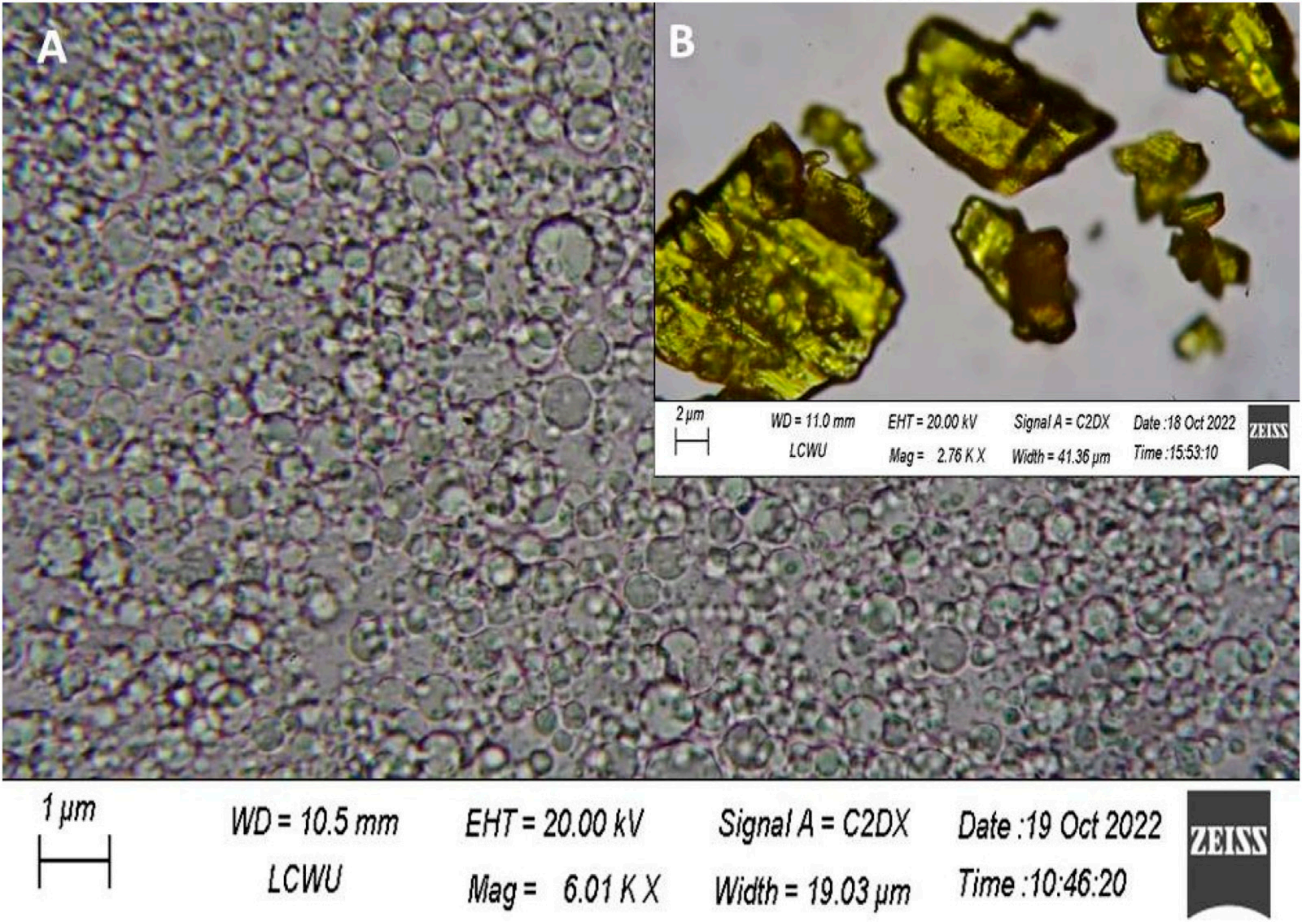
Images of pure tretinoin crystal form **(B)** and H-T-SLN particles **(A)**.

Scanning electron microscopy was used to directly observe the morphological appearance of the SLN. H-T-SLN was shown to be spherical in shape after the particles were scanned under an electron microscope. Scanning electron microscopy (SEM) micrographs of pure treatment and an optimized SLN formulation clearly demonstrated significant differences. The ordered shape of H-T-SLN may be the result of phase evaporation during particle hardening. Figure 7A shows a scanning electron microscopic view (SEM) of H-T-SLN, which showed well-dispersed particles. The results showed that successful SLN with ordered shapes were prepared from API crystalline particles, as shown in Figure 7B, which depicts large crystal particles.

### X-ray diffraction (XRD)

**FIGURE 8:**
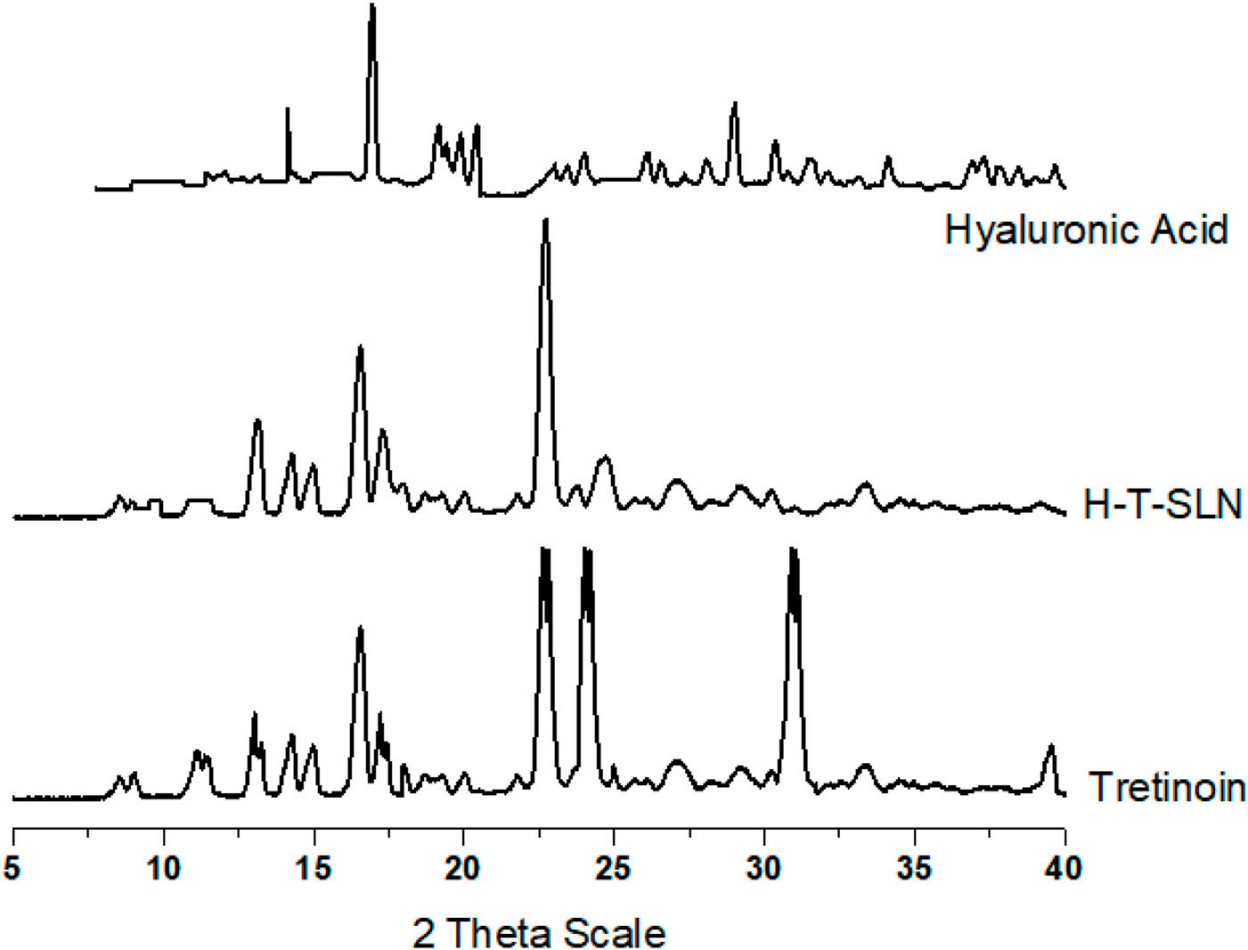
XRD patterns of pure tretinoin, hyaluronic acid and H-T-SLN.

The tretinoin diffraction pattern revealed five main distinct peaks at 16.519 2θ, 22.603° 2θ, 24.004, and 39.482 2θ, indicating its crystalline nature, as shown in Figure 8. In the diffractogram of the H-T-SLN, these peaks could not be seen except two with low intensity, indicating that the tretinoin was dissolved inside the lipid matrix of the SLN and stabilized in an amorphous state. Because of its limited solubility in water, tretinoin would crystallize if it were present outside of the lipid matrix, which should have had an impact on the diffraction patterns of drug-loaded SLN. This shows that the medication was successfully absorbed into the SLN lipid matrix. Additionally, XRD spectra between angles 15.219 2θ and 28.503° 2θ showed lower intensity in H-T-SLN than in hyaluronic acid, which indicates lower crystallinity in comparison to neat tretinoin and hyaluronic acid. This observation can be explained by the fact that tretinoin was trapped in the lipid core of SLN.

### *In vitro* release study

**FIGURE 9:**
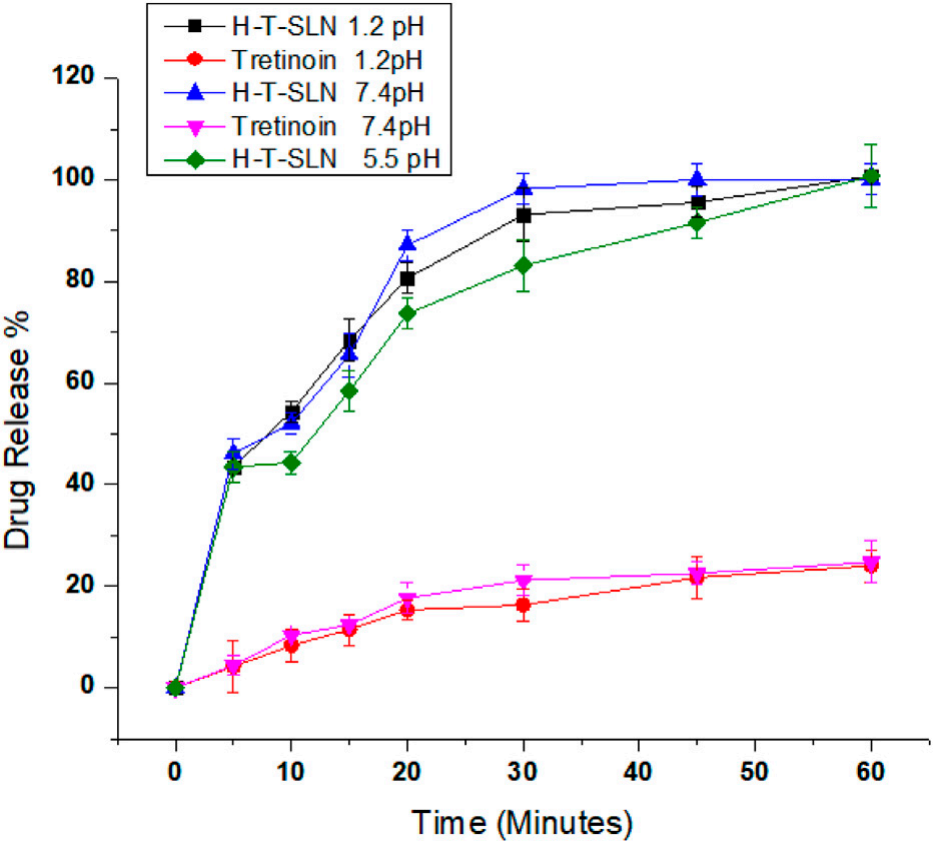
*In vitro* drug release profile of pure drug and formulation studied in acidic buffer pH 1.2, phosphate buffer pH 5.5 and 7.4 according to dialysis bag membrane method (n = 3).

Tretinoin pure medication and H-T-SLN were checked for dissolution in two buffers for up to 60 min. The release profile from both types is shown in Figure 9. The H-T-SLN particle release profile displayed an immediate release pattern that was biphasic in nature (Figure 7). The mean particle size of the H-T-SLN samples employed in this study was 750 nm (PDI-0.276), which is helpful for increasing the dissolution of H-T-SLN particles in both media, and its rapid dissolution results in immediate absorbance. The aqueous solubility of tretinoin is very low, 2% (w/v). It displayed a nearly identical pattern of low solubility in both media. The percent cumulative drug release versus time was plotted to demonstrate the drug release pattern (Figure 9). The results demonstrated that drug release from the H-T-SLN particles was very fast. The drug release after 30 min was above 85% ± 3.79% in both media. Comparative results shown by Mishra et al. (2018) in his research which was 99.78 ± 0.78 within 120 min in this they prepared ethosomes of tretinoin for acne treatment. We have also checked H-T-SLN on 5.5 pH because this is skin pH and we have observed significant release pattern on this pH 83.12% in 30 min and 100.00% in 60 min.

Burst release was observed within 10 min in both buffers, followed by a continuous drug release pattern and 100% and 97% dissolution within 60 min in both buffers. Comparative results we have found in research which was conducted by Montassier et al. (1998).

### Haemolysis investigations

**FIGURE 10:**
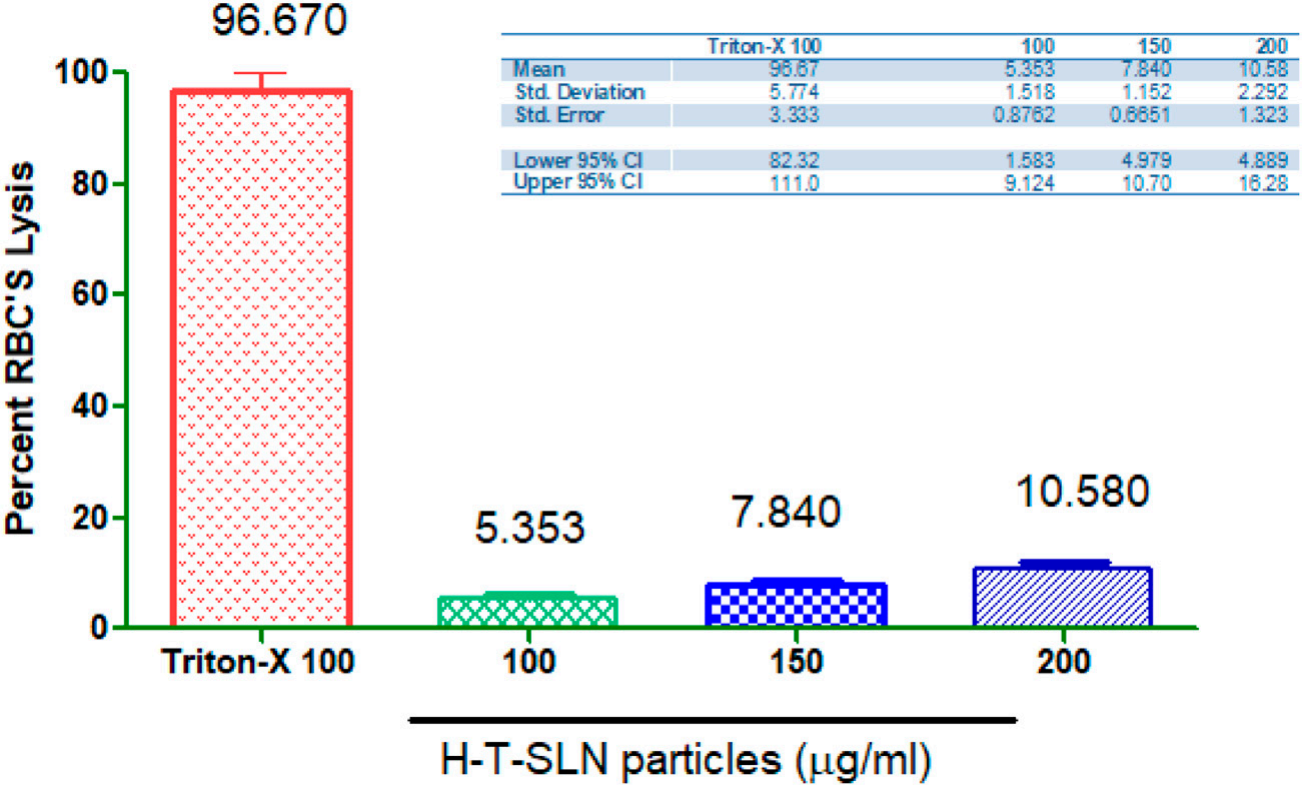
The compatibility of H-T-SLN particles with blood.

Through hemolytic studies, the compatibility of the H-T-SLN particles with the blood of rats was examined. Hemolytic analyses revealed that H-T-SLN particles did not cause hemolysis after administration at concentrations of 100, 150, and 200 mg mL−^1^; however, negligible hemolysis was noted even at high concentrations (5.353, 7.840, and 10.580%), i.e., 200 mg mL−^1^, and blood cells demonstrated good tolerance to the complex (*p* < 0.0001). Relevant study was done by Ali et al. (2018) in which he shown 15.34% ± 0.89% and 19.67% ± 1.29% hemolysis for 500 and 1,000 μg mL^–1^ concentrations of SLN. The current study’s findings showed (Figure 10) that cell viability relied on concentration and that cells were still alive after being incubated with H-T-SLN, confirming the compatibility of the substance with human blood.

### Physical stability and microbial assessment

After converting H-T-SLN into a serum formulation, we determined its stability and total aerobic microbial count (TAMC). The H-T-SLN serum formulation was developed for skin applications. Stability tests were performed for H-T-SLN using a stability chamber in which H-T-SLN was held at 40°C for 6 months. On the initial day, after 3 months and after 6 months, H-T-SLN, order, description, pH, particle size, PDI and viscosity were each examined. The pH of the H-T-SLN serum formulation was found to be 6.3 to 6.5, which is appropriate for skin products. The formulation’s viscosity at 25°C was 79 to 80 cpi, making it appropriate for skin application as well. For H-T-SLN, serum homogeneity and clarity were deemed satisfactory. Particle size and PDI was also determined after 6th month to ensure particles stability and found satisfactory. It was 750 ± 31.29, 0.24 ± 0.12 in initial and after 6th month it was 710 ± 41.12, 0.27 ± 0.22.

H-T-SLN serum must comply with all USP 43 requirements before being made into a scaled-up formulation for patients (Adriaensen et al., 2017). The microbiological parameters of H-T-SLN serum were also checked. TAMC, TYMC, *Staphylococcus*, and *Pseudomonas* were examined in H-T-SLN for up to 6 months. To prepare the plates, Sabouraud Dextrose Agar and Nutrient Agar were used, and the method was described in the publication by Ballal et al. (2009). Plates were incubated in Memmert incubators for 72 h TAMC and 7 days for TYMC at different temperatures (32℃ ± 2 and 22℃ ± 2). The results shown in Table 2 are quite satisfactory until the 6th month.

### *In vitro* permeability

**FIGURE 11:**
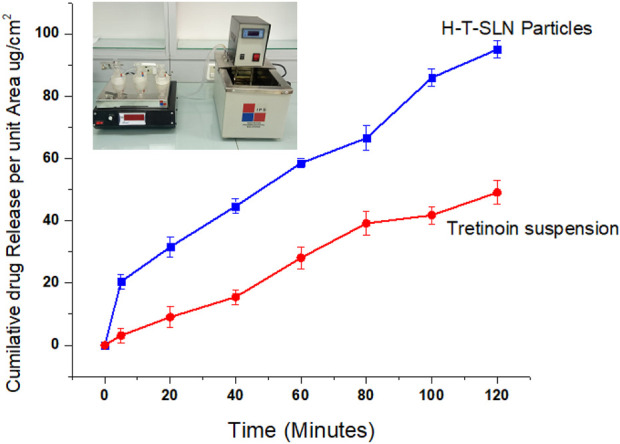
Tretinoin release in the form of a suspension through a silicon membrane compared with H-T-SLN release.

The primary goal of the current investigation was to evaluate the *in vitro* permeability of H-T-SLN over a silica membrane (pH 6.8). Clean, dry receptor cells filled with deaerated buffer were used in this investigation. The mixture was stirred continuously with a magnetic stirrer for 30 min at 37°C. To stop direct evaporation, para-film was used to plug the apertures. To stir the receptor compartment, a speed of 200 rpm was maintained. Krüger (2010) mentioned in his experiments that SLN preparation are more absorbable through skin (Krüger, 2010). Drug diffusion was measured using a prehydrated silica membrane. A 0.5 mL sample was obtained using a glass syringe from the receptor cell for UV analysis at 295 nm wavelength. Figure 11 depicts the results, which revealed that at pH 6.8, H-T-SLN diffused twice as much within a short time as pure medication. This enhancement has been made possible by the lower particle sizes obtained using the precipitation process of manufacturing SLN. The apparent permeability constant Papp was calculated according to Fick’s first law of diffusion (Reichling et al., 2006). The values for flux and apparent permeation coefficient of H-T-SLN were calculated according to Fick’s first law of diffusion. Flux of H-T-SLN through silica membrane ( μ L/cm^2^ h) mean ± SE (0.021 ± 0.002). Papp of H-T-SLN according to Fick’s first law was 2.64 ± 0.04 (cm/s). (Data of suspension is not included in manuscript).

### Skin safety evaluation

**FIGURE 12:**
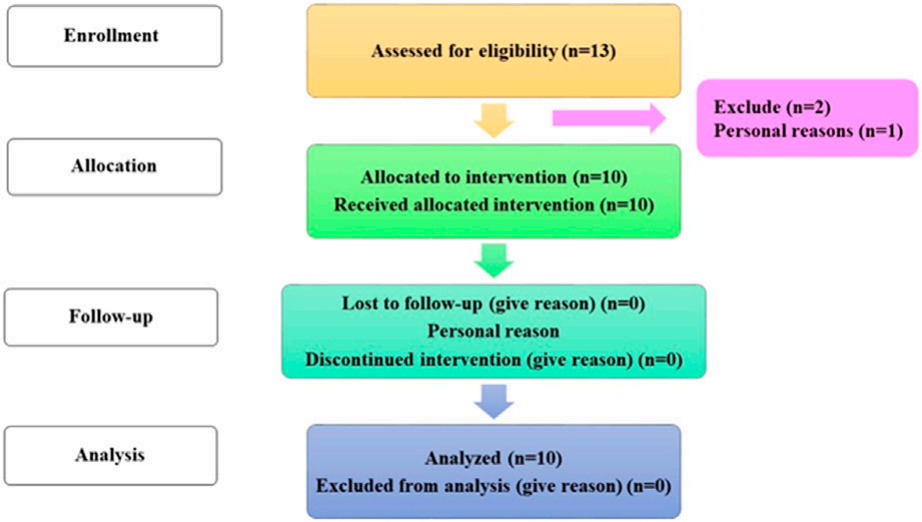
Flow chart of the subject allocation process.

For skin safety evaluation, skin irritation and skin dermatitis were viewed after applying H-T-SLN serum on the subject’s face after consent. Before applying the H-T-SLN serum on the subject’s face, it was cleaned with soap, and the skin was dried with tissue. After this, 1 g of H-T-SLN serum was gently applied to the face skin with fingers. After 24 h, the skin was observed visually and found to be healthy. No irritation or dermatitis marks appeared on the skin, which indicated that the H-T-SLN serum is safe for skin use. Follow chart shown in Figure 12.

### Visual assessment of skin wrinkles

To determine the ameliorating effect of H-T-SLN on wrinkle formation, wrinkles around the eyes of the subjects were evaluated independently under specific lighting conditions. Skin damage was determined by visual assessment, and no irritation, damage or side effects were found in any of the subjects. A 4 week study was conducted, and 60% of the subjects found a significant decrease in wrinkles with daily use of H-T-SLN.

### Evaluation of skin wrinkle parameters using Visioscan^®^ camera images

**FIGURE 13:**
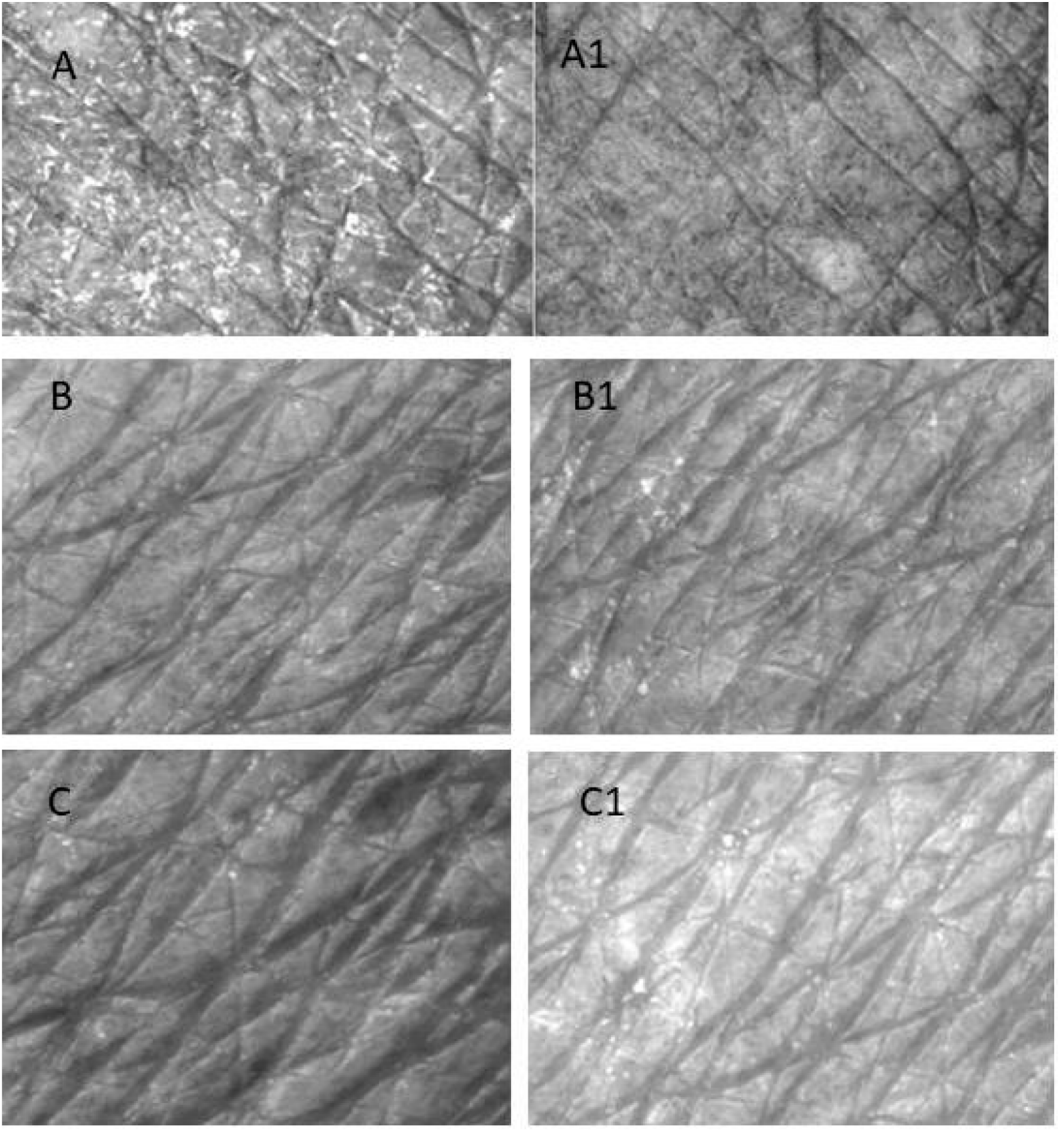
Surface of the skin of a three volunteers who applied (H-T-SLN) serum before **(A–C)** 4 weeks of application. The skin surface was evaluated by Visioscan^®^ VC98 software after 4 weeks **(A1–C1)**.

The objective of this study was to evaluate through objective and subjective analysis the long-term effects of H-T-SLN serum containing tretinoin and hyaluronic acid. H-T-SLN was applied to the face skin of 10 volunteers (skin pictures of three voluntaries are shown in Figure 13). Skin smoothness (Se), skin roughness (Se), scaliness (Se) and wrinkles (Se) were determined after 4 weeks of daily application. One of the volunteer skin surfaces is shown in Figure 13. The results showed significant changes in parameters such as skin tightness, which may be produced by surface deposition of certain film-forming polymers and tretinoin. Table 3 shows all volunteers’ skin smoothness (Se), skin roughness (Se), scaliness (Se) and wrinkles (Se). The moisturizing effects (glycerin) could, in addition, reduce the irritant effects of anti-aging treatments such as those with tretinoin.

The clinical portion of our investigation involved ten participants with mild to advanced wrinkles (Table 3) who were both sexes and between the ages of 40 and 60 (mean age, 40 ± 8.76 years). There were no significant side effects seen, and there were no reports of skin erythema, burning, itching, or peeling throughout the course of treatment. Ninety percent of individuals improved by at least one grade after 4 weeks of treatment, according to the subjective efficacy evaluation (Table 4). Additionally, after the procedure, the volunteer reported feeling “moderately happy.” In both follow-up visits, there was a noticeable reduction in the volume and wrinkle area of the nasolabial folds according to objective measurements. In the fourth week, the wrinkle area decreased from 41–66 (baseline) to 37–6. Finally, there was no discernible change in the moisture of the skin.

### Critical quality attributes (CQAs)

**FIGURE 14:**
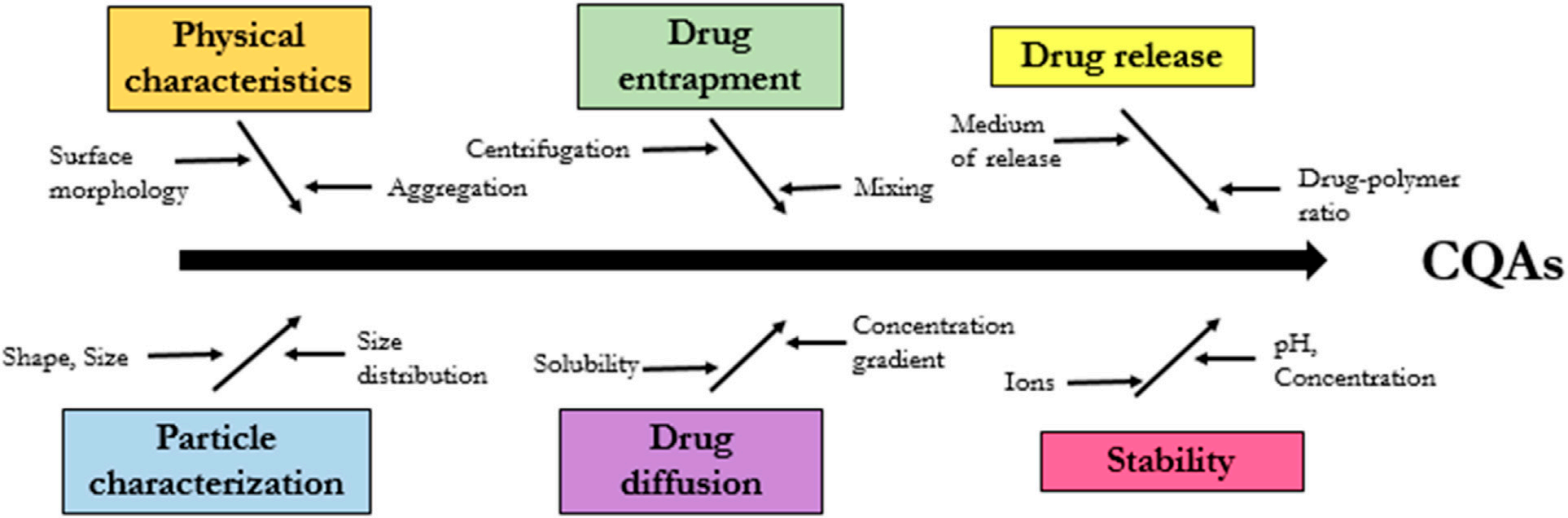
Critical Quality Attributes (CQAs) Fish bone diagram.

The FDA issued guidelines for the industry in 2018 regarding the development of SLN particles medicinal products, citing a number of crucial quality attributes (CQAs) that require attention. These CQAs are shown in Figure 14 and include: physical characteristics, particle size, drug entrapment, drug diffusion and drug release and stability structure. We cover here the methodologies and technologies that have been used to describe SLN pharmaceutical formulations, with an emphasis on CQAs, in an effort to better characterize and successfully develop these formulations.

## Conclusion

SLN nanotransporters are efficient carriers for drug delivery to the skin. Due to the superior ratio of particle surface to volume, small sized SLN nanotransportare most promising for skin delivery. This mechanism even surmounts the effect of mixing of epidermal lipids and the matrix lipids of SLN, which can facilitate penetration especially of drugs. Hyaluronic acid based SLN with tretinoin loading were developed for topical delivery. H-T-SLN was shown to have been converted to an amorphous state by DSC and XRD tests. Hemolysis assay shown that H-T-SLN is compatible rat blood and there is no toxic effect of H-T-SLN. The improved H-T-SLN formulation remained stable for over 6 months at 40℃. Further we have converted it into serum form for easy applications. In conclusion, this investigation showed H-T-SLN serum resulted in wrinkles reduction in the human. In addition, the daily use of these substances is very important to protect the barrier function of the skin, The immediate effects observed in the short term study were confirmed in the long term evaluation.

## Data Availability

The raw data supporting the conclusions of this article will be made available by the authors, without undue reservation.
